# ﻿*Xylochironomus* Cranston newly recorded in the Oriental region (Diptera, Chironomidae), with description of three new species

**DOI:** 10.3897/zookeys.1257.163285

**Published:** 2025-10-29

**Authors:** Shutang Liu, Hongqu Tang

**Affiliations:** 1 Research Centre of Hydrobiology, Jinan University, Guangzhou 510632, China Jinan University Guangzhou China

**Keywords:** Emendation, first record, key, new species, Oriental region, taxonomy

## Abstract

Three new species of *Xylochironomus* Cranston are described here based on adult males from the Oriental region, specifically from South China and East Malaysia. The generic diagnosis for the adult male is emended, and a key to the males of known species is provided. This is the first formal record of *Xylochironomus* from the Oriental region, and indeed the first occurrence outside the Australian region, providing another representative in the Gondwana biogeographic clade.

## ﻿Introduction

*Xylochironomus* Cranston is a monotypic genus based on northern Australian material, with the type species *Xylochironomus kakadu* Cranston (Cranston 2006). As one of the typical wood-mining chironomids, the adult and larva which are subject to different evolutionary pressures, show two different morpho-responses and evolutionary pathways. The adult male resembles those Polypedilum (Pentapedilum), while the larva is clearly distinct, e.g., the orientation and shape of the ventromental lobe and mandible, which is associated with a mining immersed-wood life. Similar larvae were found subsequently in Thai streams (Sriariyanuwath et al. 2015), and they were noted in the South East Asia guide (Cranston and Tang 2024). However, no adult species has been reported to date in this region. During the diversity exploration in Southeast Asia and South China in recent years, several unusual species of subgenus “*Pentapedilum*” formed an independent clade when testing the molecular phylogeny using multi-gene makers, closely nested with the Australian *Xylochironomus kakadu* (voucher no.: AUNT04), and then the *Endochironomus* complex and *Stenochironomus* complex, rather than the presumed *Polypedilum* complex (personal unpublished data). Further examination of the morphological characters reveals that all belong to the genus *Xylochironomus* Cranston, and all are new to science. Here, we describe *X. meng* sp. nov., *X. mulu* sp. nov. and *X. yue* sp. nov., and provide some generic emendations.

## ﻿Material and methods

Adults were collected using light traps or sweeping nets. All specimens were slide-mounted in Euparal. Photographs were taken of each specimen under an Olympus BX53 compound microscope using a mounted camera (ToupView^TM^). Digital photographs of different focal planes were stacked using Helicon Focus version 7. Line drawings were aided by the use of a drawing tube attached to an Olympus BX53. Morphological terminology and abbreviations largely follow Sæther (1980). Measurements are given as ranges, with the number of observed specimens in parentheses if different from the number (*N*) stated at the beginning of the description. All material is deposited in the Department of Ecology, Research Centre of Hydrobiology, Jinan University, Guangzhou, Guangdong.

### ﻿Genus Xylochironomus

Taxon classificationAnimaliaDipteraChironomidae

Cranston, 2006

E314F5B8-E9BC-5B3F-A591-79DB63933EC6

#### Type species.

*Xylochironomus kakadu* Cranston, 2006.

#### Diagnosis.

The specimens examined conform in most diagnostic features to the generic description of the adult male (Cranston 2006). Based on the material described below, the generic diagnosis for *Xylochironomus* should be emended as follows.

#### Adult male.

**Figure 1.**
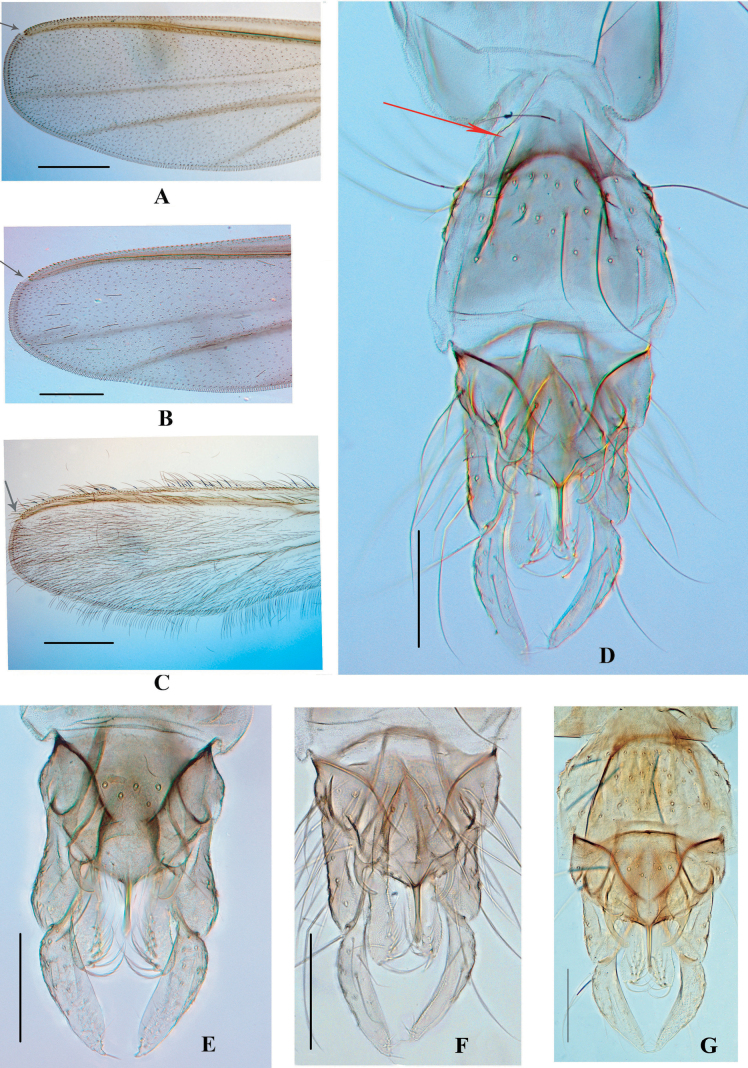
Color photos of wing apex (A–C, arrows indicate the costa disjunction), abdominal terminal (D, arrow indicates the constricted anterior TVIII) and male genitalia (E–G). A, E. *X. meng* sp. nov.; B, D, F. *X. mulu* sp. nov.; C, G. *X. yue* sp. nov. Scale bars: 200 µm (A–C); 100 µm (D–G).

**Figure 2.**
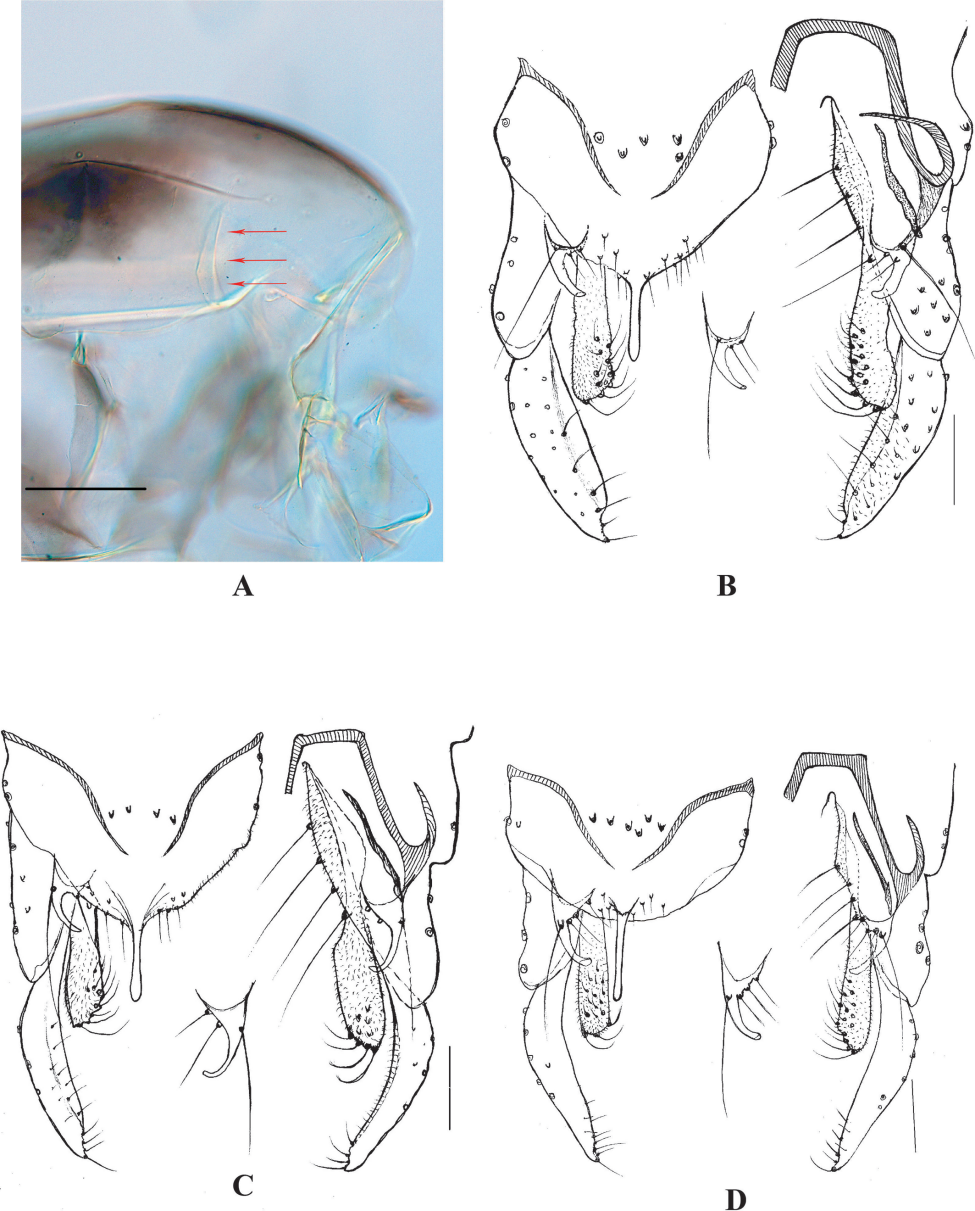
Color photos of *Xylochironomus* thorax (A) and line drawings of genitalia (B–D). A, B. *X. meng* sp. nov., arrow indicates the thick parapsidal suture; C. *X. mulu* sp. nov.; D. *X. yue* sp. nov. Scale bars: 100 µm (A); 50 µm (B–D).

Frontal tubercles small, reduced. Acrostichals biserial, starting from the anterior scutum. Parapsidal suture thick (Fig. 2A). Scutal hump with a weak hump, very low. Wing cuneiform and setose, anal lobe clearly reduced, apex of wing somewhat rectangular, with an interrupted costal extension (Fig. 1A–C). Squama bare or with fewer than 3 setae. Mid and hind tibiae with 2 separated combs, only the outer comb with a long-curved spur, the length ratio of ta4/ta5 in the fore and hind leg usually no less than 2.0. Segment VIII tapered anteriorly (Fig. 1D) or indistinct in some teneral form. Insertion of lateral seta of superior volsella arising basal-dorsally or from basal margin; outer seta usually much longer than the length of the superior volsella. Inner margin setae of gonostylus short and sparse, unequal-distance or irregular. Anal tergite band separated, anal median seta tends to be uniserial if less than 6 setae, no tendency to be enclosed by a round and sclerotized anal tergite circle.

#### Adult female, pupa and larva.

See Cranston (2006).

### ﻿ Xylochironomus meng sp. nov.

Taxon classificationAnimaliaDipteraChironomidae

DB099F96-B628-5384-BC8D-6A65D4969E16

https://zoobank.org/B89E5217-9295-4FB4-BA2B-C24CC521E20B

Figs 1A, 1E, 2A, B

#### Material examined.

***Holotype***: M, China • Yunnan Province, Pu’er City, Ximeng Wa Autonomous County, Longtan Pool in Mengsuo Town, 22°38'N, 99°35'E, alt. 1,917 m, light traps, 27.viii.2014, HQ Tang. ***Paratype***: • 1M, same as holotype except 26.iv.2022.

#### Diagnosis.

The new species is characterised by the gonostylus with an acute apex and high leg value (LR_1_ 2.2–2.4).

#### Etymology.

For the type locality, “*meng*” is a term used to refer to certain places in Yunnan dialect, similar to a town or county. The name is a noun in apposition.

#### Description.

Male imago (*N* = 2).

Total length 2.8–3.3 mm. Wing length 1.6–1.7 mm. Total length/Wing length 1.71–1.97.

***Head*.** Antennal terminal flagellomere 460–490 μm long, AR 0.72–0.80; apex with 2–3 long terminal setae, 85–110 μm long. Frontal tubercle ca. 2–3 μm long. Temporals with 4–6 verticals. Clypeus with 15–21 setae. Palpomeres 1–5 (in μm): 25–30; 50–55; 110–130; 110–125; 200–240 in length.

***Thorax*.** Acrostichals 10–12; dorsocentrals 8–10; prealars 3–4; scutellars 8–10.

***Wing*.** VR 1.16–1.21. R with 45–55 setae; R_1_ with more than 50 setae; R_4+5_ with more than 150 setae. Square 2–3 setae.

***Legs*.** Scale of fore tibia round, 25–35 μm long, spurs of mid tibia and hind tibia 35–55 μm and 50–65 μm long, respectively. Lengths and proportions of legs as Table 1.

**Table 1.** Lengths (in µm) and proportions of leg segments of *Xylochironomus meng* sp. nov., male (*N* = 2).

	fe	ti	ta1	ta2	ta3	ta4	ta5	LR
P1	850–1000	430–550	1120–1225	775–800	500–510	425–440	170–180	2.23–2.40
P2	820–985	680–825	430–500	250–290	170–210	110–140	60–70	0.59–0.63
P3	870–1000	720–828	570–700	320–375	290–350	190–225	80–100	0.79–0.85

***Hypopygium*** (Figs 1E, 2B). Tergite IX with 5–8 median setae, arranged in one or two rows. Anal point parallel, 45–48 μm long, not reaching the apex of inferior volsella. Gonocoxite 150–165 μm long, superior volsella 40–50 μm long, with two basal inner setae and one additional (‘outer’) seta, arising from the basal margin of superior volsella, 75–80 μm long, extending near the apex of inferior volsella. Gonostylus 120–125 μm long, with acute apex. HR 1.28–1.38.

#### Remarks.

The new species somewhat resembles the Okinawa species Polypedilum (Pentapedilum) acristylum Yamamoto, Hirowatari & Yamamoto, 2012 (Yamamoto et al. 2012). Both have an acute apex to the gonostylus, and the location of the outer seta of the superior volsella is more basal. However, in *P. acristylum*, the outer seta of the superior volsella is relatively short, not extending beyond the apex of the superior volsella, the squama has 9 setae, and the ratio of ta4/ta5 in the middle leg is 1.5, and thus *P. acristylum* is not a member of *Xylochironomus*, but an atypical species of Polypedilum (Pentapedilum).

### ﻿ Xylochironomus mulu sp. nov.

Taxon classificationAnimaliaDipteraChironomidae

F21B3658-7E15-5E01-9956-784E556C1E0D

https://zoobank.org/52682106-1548-4F4D-A02E-286B2DBA8071

Figs 1B, 1F, 2C

#### Material examined.

***Holotype***: M, Malaysia • Sarawak, Gunung Mulu National Park, Melinau stream, Paku tributary, alt. 75 m, 04°1'N, 114°49'E, light traps, 12.vi.2023, leg. H.Q. Tang. ***Paratype***: • 1M as holotype.

#### Diagnosis.

The new species is characterised by the presence of a small frontal tubercle and the contour of the superior volsella.

#### Etymology.

From the type locality, Gunung Mulu National Park. The name is a noun in apposition.

#### Description.

Male imago (*N* = 2).

Total length 2.8–3.2 mm. Wing length 1.4–1.5 mm. Total length/Wing length 1.93–2.19.

***Head*.** Frontal tubercle small, 5–6 μm wide and 6–10 μm long. Antenna terminal flagellomere 420–450 μm long, apex with 2–3 long apical setae, 80–100 μm long. AR 0.79–0.84. Temporals with 8–12 inner verticals. Clypeus with 18–21 setae. Lengths of palpomeres 1–5 (in μm): 30–33; 45–50; 100–110; 120–125; 190–210.

***Thorax*.** Acrostichals 16–20; dorsocentrals 8–9; prealars 3; scutellars 5–7 biserial setae, anterior row with 2–4 small setae medially.

***Wing*.** VR 1.18–1.20. R, R_1_ and R_4+5_ with more than 35 setae. Squama with 0–2 setae.

***Legs*.** Scale of fore tibia tongue-shaped, 25–30 μm long; spurs of mid tibia and hind tibia 60–70 and 55–70 μm long, respectively. Lengths and proportions of legs as Table 2.

**Table 2.** Lengths (in µm) and proportions of leg segments of *Xylochironomus mulu* sp. nov., male (*N* = 2).

	fe	ti	ta1	ta2	ta3	ta4	ta5	LR
P1	1000–1050	510–540	n/a	n/a	n/a	n/a	n/a	n/a
P2	900–1050	750–825	625–670	325–360	300–330	200–220	100–110	0.81–0.83
P3	870–880	720–800	440–490	250–270	180–190	110–140	60–70	0.61–0.62

***Hypopygium*** (Figs 1F, 2C). Tergite IX with 4–6 median setae in one row. Anal point 50–55 μm long, extending to the subapex of inferior volsella. Gonocoxite 150–155 μm long, superior volsella with two basal inner setae arising from the distinct tubercles, and one outer seta, arising from the basidorsal surface of superior volsella, 70–80 μm long, extending the apex of inferior volsella. Gonostylus 115–120 μm long. HR 1.25–1.30.

#### Remarks.

The new species is characterized by the relative location between inner and outer seta of the superior volsella, and the outer seta extending beyond the terminals of the inferior volsella. The shape of the gonostylus seems to vary depending on orientation.

### ﻿ Xylochironomus yue sp. nov.

Taxon classificationAnimaliaDipteraChironomidae

6E362E93-F89D-58C4-9D01-BBD1816CE70C

https://zoobank.org/FC5A8FAA-572B-40D0-B29A-3AB068971961

Figs 1C, 1G, 2D

#### Material examined.

***Holotype***: M, China • Guangdong Province, Guangzhou City, Conghua District, Lyutian Town, Fentian, 23°50'N, 113°56'E, alt. 260 m, light traps, 02.iv.2018, leg. HQ Tang. ***Paratype***: • 1M, as holotype.

#### Diagnosis.

The new species can be separated from congeners by the large wing length (c. 2.0 mm), wing squama with 1–3 setae, and the outer seta of the superior volsellae distinctly extending beyond the apex of the superior volsellae.

#### Etymology.

For the type locality, “*yue*” is an abbreviation of Guangdong Province. The name is a noun in apposition.

#### Description.

Male imago (*N* = 4).

Total length 3.0–3.2 mm. Wing length 1.8–1.9 mm. Total length/Wing length 1.68–1.75.

***Head*.** Frontal tubercle present, ca. 5 μm long. Antenna terminal flagellomere 510 μm (*N* = 1) long. AR 0.80 (*N* = 1). Temporals with 10–12 inner verticals. Clypeus with 18–21 setae. Lengths of palpomeres 1–5 (in μm): 30–35; 40–45; 120–125; 125–130; 235–240.

***Thorax*** (*N* = 1). Acrostichals 14; dorsocentrals 8; prealars 3; scutellars 9, uniserial.

***Wing*.** VR 1.18–1.20. R, R_1_ and R_4+5_ with more than 40 setae. Squama with 1–3 setae.

***Legs*.** Scale of fore tibia 30 μm long; spurs of mid tibia and hind tibia 35–45 μm and 50–60 μm long, respectively. Lengths and proportions of legs as Table 3.

**Table 3.** Lengths (in µm) and proportions of leg segments of *Xylochironomus yue* sp. nov., male (*N* = 2).

	fe	ti	ta1	ta2	ta3	ta4	ta5	LR
P1	1020–1030	540–560	1300	800	540	450	180	2.32
P2	1000–1060	800–880	510–540	280–290	200–210	120–130	60–70	0.61–0.64
P3	1025–1050	850–900	720–730	390–400	340–350	220–230	95–100	0.80–0.86

***Hypopygium*** (Figs 1G, 2D). Tergite IX with 6–10 median setae, arranged in one row, and 4–6 margin setae on each side. Posterior margin of TIX somewhat smooth. Anal point parallel, 60 μm long, extending not beyond the inferior volsella. Gonocoxite 150–155 μm long, superior volsella 55–60 μm long, with three basal inner setae and one outer seta 80–85 μm long, extending to the apex of inferior volsella slightly. Gonostylus 120–130 μm long, with attenuated apex. HR 1.20–1.25.

#### Remarks.

This new species resembles *Xylochironomus kakadu* Cranston in the shape of the posterior margin of TIX and superior volsella, while the outer seta of the superior volsella is different, clearly extending beyond the terminal of the superior volsella in this new species, but relatively shorter in the latter, less than the whole length of the superior volsella.

### ﻿Key to all known species of adult males of *Xylochironomus* Cranston, 2006

**Table:** 

1	Wing membrane strongly hirsute, with some stable macrotrichiae (Fig. 1C); length of superior volsella subequal or slightly shorter than the anal point	***X. yue* sp. nov. (China, Guangdong)**
–	Wing membrane microtrichiose, only some setal pits remain (Fig. 1A, B); length of superior volsella clearly shorter than the anal point	**2**
2	Posterior margin of TIX somewhat smooth or truncated. Wing squama bare. Foreleg ratio more than 2.5	***X. kakadu* Cranston (N. Australia)**
–	Posterior margin of TIX triangular. Wing squama usually with 1–3 setae. Foreleg ratio less than 2.5	**3**
3	Outer seta of superior volsella arising from the inner basal margin (Fig. 2B); gonostylus with an acute apex	***X. meng* sp. nov. (China, Yunnan)**
–	Outer seta arising from basal section of superior volsella (Fig. 2C); gonostylus with an attenuated apex	***X. mulu* sp. nov. (Malaysia, Sarawak)**

## ﻿Discussion

Generally, *Xylochironomus* Cranston can be separated from other similar genera by the reduced frontal tubercle and weak scutal hump, a few squama setae in the wing with somewhat rectangular apex and the peculiar location of the outer seta of the superior volsellae. Although the characters used in the generic diagnosis and key are summarized from limited slides, they are sufficient to separate the present species; however, there is no guarantee of reliability when further species are reported in the future. The most distinct generic characters come from those pupa and larva, such as the broken hook row on pupal tergite II, and the inclined larval ventromental plate. Hence, the associated material is most important when no molecular data is available.

The new findings of male *Xylochironomus* from Eastern Malaysia and South China, together with the larval report from Thailand (Sriariyanuwath et al. 2015), is an example of another Gondwanan clade in the Oriental region, which has already been reported in *Conochironomus* Freeman, 1961 (Cranston 2016), *Skusella* Freeman, 1961 (Cranston and Tang 2018), *Paraskusella* Cranston, 2018 and *Kribiodosis* Kieffer, 1921 (Cranston 2018; Han et al. 2021; Tang and Cranston 2025). This distribution pattern is discussed as the tropical Gondwana track by Cranston (2005). More representatives, including their immature stages and molecular phylogenetic studies, will be necessary to further explore this pattern.

## Supplementary Material

XML Treatment for Xylochironomus

XML Treatment for Xylochironomus meng

XML Treatment for Xylochironomus mulu

XML Treatment for Xylochironomus yue

## ﻿Additional information

### Use of AI

No use of AI was reported.

### Author ORCIDs

Shutang Liu https://orcid.org/0009-0008-7283-4932

Hongqu Tang https://orcid.org/0000-0002-4957-4617

### Data availability

All of the data that support the findings of this study are available in the main text.
